# Effects of Additional Flexible and Rigid Structure on BDT-BDD Terpolymer and the Performance of Organic Solar Cells

**DOI:** 10.3390/polym17020248

**Published:** 2025-01-20

**Authors:** Xin Jing, Xuebing Li, Yong Zhao, Quanliang Wang, Xiao Kang, Xiaojie Liu, Aziz Saparbaev, Feng Li, Mingliang Sun

**Affiliations:** 1 Analytical and Testing Center, Qingdao University of Science and Technology, Qingdao 266042, China; jingxin@stu.ouc.edu.cn; 2 School of Materials Science and Engineering, Ocean University of China, Qingdao 266100, China; y.zhao1972@foxmail.com (Y.Z.); wql@stu.ouc.edu.cn (Q.W.); kangxiao1994@126.com (X.K.); liuxiaojie19921005@163.com (X.L.); 3 Chemistry Department, Paderborn University, 33098 Paderborn, Germany; lixuebing2121@163.com; 4 Institute of Ion-Plasma and Laser Technologies, Uzbekistan Academy of Sciences, 33, Durmon yuli, Tashkent 100125, Uzbekistan; saparbaevaziz83@gmail.com

**Keywords:** flexible and rigid structures, terpolymers, conformation adjustment, organic solar cells

## Abstract

In organic solar cells, the aggregation and crystallization of polymers are significant for bulk heterojunction. Blending with acceptor materials, polymer donor materials can adjust their aggregation by the movement of the chain segments. In this paper, the unfused structures based on thiophene and carbazole are respectively designed and introduced into the donor-acceptor copolymer donor materials to investigate the influence of flexible and rigid structures on polymer-aggregation leading photoelectric performance. The material and quantum chemical property investigations show that the selection and design of the blocks are important for the properties of the terpolymers, and the resulting polymer:Y6 devices achieve improvements in performance from 13.85% to 15.66% (especially for fill factors from 63.37% up to 69.81%). This result contributes to designing and optimizing efficient polymers.

## 1. Introduction

For bulk heterojunction (BHJ) organic solar cells (OSCs), the organization and morphology of the active layer are intimately related to the photoelectric performance [1,2,3]. Current popular non-fullerene small-molecule acceptor materials, whether ITIC or Y6-series, often need to be finely optimized for forming a reasonable active material stacking or crystallization, significantly improving device performance [4]. For example, thermal annealing of the active layers can promote the phase segregation of the donor and acceptor materials to achieve the optimal morphology of the BHJ, while solvent evaporation treatment is to better self-organize the materials in nanodomains [5]. However, controlling requires careful attention. On the one hand, insufficient or poor aggregation behavior will suppress exciton separation by poor phase separation [6]. On the other hand, excessive phase separation also leads to excessive crystalline regions, resulting in reduced donor-acceptor material interfaces and poor OSC performance [7]. Therefore, reasonably optimizing the active layer to function in the best state is significant in OSC research.

The general approach to BHJ optimization includes three channels. Firstly, pre-treatment of the BHJ-forming solution, including volatile additive incorporation, ternary blending, heat or cold treatment of the film-forming solution, etc. Secondly, post-treatment of the BHJ active layer, such as thermal annealing, solvent evaporation treatment, etc., and thirdly, optimizing molecule structures, i.e., modifying active materials molecule structures to retain the light absorption properties and improve the aggregation and crystallization properties. BHJ film forms in a non-equilibrium morphology within several seconds, molecule conformations and leading self-organized aggregation are frozen. Thus, morphology modification depends on polymer-chain mobility [8]. No matter the methods, the aim is to adjust the molecular conformation by changing the conditions, which regulates aggregation by balancing molecular interactions. However, adjusting the molecular conformation and regulating molecular aggregation need enough energy to turn an appropriate aggregation morphology, which is decided by chemical structures. Thus, optimizing molecule structures is important.

Optimizing molecule structures can fundamentally solve the problem of active layer morphology, which avoids the tedious processes involved in pre-treatment or post-treatment in the industry. In addition to branched-chain engineering of molecules, modifying the molecular backbone can also have unexpected effects. For example, by linkage engineering, the conformations of terminal-linked dimers can invert from rigid to flexible, which has a distinct influence on photovoltaic performance [9]. For donor polymers, the backbones are controlled by the structural units. For example, thiophene and benzene are the two most fundamental blocks along polymer chains; however, thiophene-thiophene linkages tend to exhibit higher planarity than benzene-benzene linkages [10]. Furthermore, polymer crystallization can be adjusted by randomly distributing irregular structural units along the sequence of the chain [11]. The intrinsic structural properties of polymer chains, such as different degrees of conformational and configurational freedom and chain stiffness, determine how crystalline or amorphous the polymeric materials are intrinsically and how important kinetic factors are in impacting ordering in specific fabrication procedures [12]. From this point, random terpolymers can combine the local conformation regulating and the excellent binary polymer properties into the polymer backbone by optimizing structural units [13]. Flexible or rigid, structural units will improve polymer conformations, which influences the self-assembled block and the lead crystalline of polymers.

In this paper, with ester-substituted thiophene as π-bridges, the unfused units (3T and 2TKZ) based on thiophene and carbazole were designed, respectively. Composed of benzo[1,2-b:4,5-b0]dithiophene (BDT) and benzo-[1,2-c:4,5-c0]dithiophene-4,8-dione (BDD) blocks, polymers with BDT-BDD backbones exhibit strong aggregation behavior [14]. Oligothiophenes are the most frequently used material due to their flexibility and high charge-carrier mobility. The mobility of thiophene-based copolymers can be improved by incorporating a rigid fused unit, which provides a polymer with higher crystallinity and a more ordered morphology [15,16]. Thus, the 3T and 2TKZ are introduced into BDT-BDD chains and the influence of flexible and rigid structures on polymer-aggregation of synthesized terpolymers is investigated. It finds that the type and content of introduced structures can regulate polymer aggregation. As a result, the photovoltaic performance of terpolymers is improved. In 3T-terpolymer-based OSC devices, the power conversion efficiency (PCE) can improve from 13.85% to 15.66%, especially in fill factor (FF, from 63.37% to 69.81%). It demonstrates the significance of optimizing flexible and rigid molecule structures for bulk heterojunction (BHJ).

## 2. Materials and Methods

### 2.1. Materials and Synthesis

Monomers such as (4,8-bis(4-chloro-5-(2-ethylhexyl)thiophen-2-yl)benzo[1,2-b:4,5-b’]dithiophene-2,6-diyl)bis(trimethylstannane) (BDT-Sn(Me)_3_), 1,3-bis(5-bromothiophen-2-yl)-5,7-bis(2-ethylhexyl)-4H,8H-benzo[1,2-c:4,5-c’]dithiophene-4,8-dione (BDD-Br), and 2-bromothiophene-3-carboxylic acid are purchased (Derthon Optoelectronic Materials Science Technology Co., Ltd., Shenzhen, China) and used without further purification.

**Decyl 2-bromothiophene-3-carboxylate (A)**

2-bromothiophene-3-carboxylic acid (2.07 g, 10 mmol), dicyclohexylcarbodiimide (2.27 g, 11 mmol), and 4-dimethylaminopyridine (0.5 g, 4.1 mmol) were dissolved in DCM (10 mL), followed by the addition of n-decanol (2.37 g, 15 mmol) and stirring at room temperature for 50 h. After reaching the reaction time, the organic phase was collected by adding water (100 mL) and extracting it with DCM. The organic phase was dried over anhydrous Na_2_SO_4_ and the solvent was removed using a rotary evaporator to obtain crude compound A. Crude compound A was purified by silica gel column chromatography using PE/DCM as eluent (PE/DCM = 6/1, *v*/*v*). The final colorless and clear liquid A was obtained. (2.74 g, yield 79%).

^1^H NMR (600 MHz, CDCl_3_) δ 7.37 (d, 1H), 7.21 (d, 1H), 4.28 (t, 2H), 1.74 (m, 2H), 1.43 (m, 2H), 1.26 (m, 12H), 0.88 (t, 3H).

**Didecyl [2,2′:5′,2″-terthiophene]-3,3″-dicarboxylate (B)**

Compound A (1.04 g, 3 mmol), 2,5-bis(trimethylstannyl)thiophene (0.61 g, 1.5 mmol), and Pd(PPh_3_)_4_ (0.17 g, 0.15 mmol) were dissolved in dry toluene (15 mL) under N_2_ protection and stirred at reflux for 48 h. After reaching the reaction time, the heating was stopped and the reaction was cooled down naturally to room temperature. After reaching the reaction time, the heating was stopped and cooled down naturally to room temperature. The solvent was removed from the mixture using a rotary evaporator. The crude compound B was purified by silica gel column chromatography using PE/DCM (PE/DCM = 4/1, *v*/*v*) as eluent. The obtained colorless and clear liquid B. (0.81 g, yield 87%).

^1^H NMR (600 MHz, CDCl_3_): 7.50 (d, 2H), 7.39 (s, 2H), 7.21 (d, 2H), 4.24 (t, 4H), 1.68 (m, 4H), 1.24 (m, 28H), 0.87 (t, 6H).

**Didecyl 5,5″-dibromo-[2,2′:5′,2″-terthiophene]-3,3″-dicarboxylate (C)**

Compound B (0.62 g, 1 mmol) was dissolved in CHCl_3_ (20 mL) under N_2_ protection and protected from light, followed by the addition of NBS (0.45 g, 2.5 mmol) to the solution. The reaction was carried out under stirring conditions at room temperature and protected from light for 48 h. After reaching the reaction time, the organic phase was collected by adding water (100 mL) and extracted with DCM. The collected organic phase was dried with anhydrous Na_2_SO_4_. Finally, the solvent was removed using a rotary evaporator to obtain crude compound C. Crude compound C was purified by silica gel column chromatography using PE/DCM (PE/DCM = 3/1, *v*/*v*) as eluent. A yellow solid C was finally obtained. (0.71 g, yield 92%).

^1^H NMR (600 MHz, CDCl_3_): 7.44 (s, 2H), 7.34 (s, 2H), 4.22 (t, 4H), 1.67 (m, 4H), 1.25 (m, 28H), 0.88 (t, 6H).

**Didecyl 2,2′-(9-(2-ethylhexyl)-9H-carbazole-2,7-diyl)bis(thiophene-3-carboxylate) (D)**

Compound D was synthesized in the same way as B. Only 2,5-bis(trimethylstannyl)thiophene was replaced by 9-(2-ethylhexyl)-2,7-bis(trimethylstannyl)-9H-carbazole and the same molar was used for each reactant in the synthesis. (0.96 g, yield 79%)

^1^H NMR (600 MHz, CDCl_3_): 8.08 (d, 2H), 7.56 (d, 2H), 7.52 (s, 2H), 7.37 (d, 2H), 7.28 (d, 2H), 4.18 (m, 2H), 4.12 (t, 4H), 2.10 (m, 1H), 1.48 (m, 4H), 1.25 (m, 36H), 0.86 (m, 12H).

**2,8-Dibromo-10,11-bis(2-ethylhexyl)-10,11-dihydro-[1,2,5]thiadiazolo[3,4-e] thieno[2′,3′:4,5]pyrrolo[3,2-g]thieno[3,2-b]indole (E)**

Compound E is synthesized in the same way as C. Only B was replaced by D and the same molar was used for each reactant in the synthesis. (0.81 g, yield 83%)

^1^H NMR (600 MHz, CDCl_3_): 8.29 (s, 2H), 7.61 (d, 2H), 7.40 (s, 2H), 7.36 (d, 2H), 4.10 (d, 2H), 4.04 (t, 4H), 2.00 (m, 1H), 1.57 (m, 4H), 1.24 (m, 36H), 0.86 (m, 12H).

**SFZn-x**

Terpolymers SFZn-x were fabricated using an identical process except for the acceptor monomer. SFZ1-0.2 was synthesized by BDT-Sn(Me)_3_ (0.1 mmol, 97.3 mg), BDD-Br (0.08 mmol, 61.3 mg), and monomer C (0.02 mmol, 15.5 mg). SFZ1-0.4 was synthesized by BDT-Sn(Me)_3_ (0.1 mmol, 97.3 mg), BDD-Br (0.06 mmol, 46.0 mg), and monomer C (0.04 mmol, 30.1 mg). SFZ2-0.2 was synthesized by BDT-Sn(Me)_3_ (0.1 mmol, 97.3 mg), BDD-Br (0.08 mmol, 61.3 mg), and monomer E (0.02 mmol, 19.4 mg). Monomers were dissolved in toluene (10 mL). Pd(PPh_3_)_4_ (20 mg) was added to the mixtures. The reactions were stirred at 110 °C for 48 h under N_2_. The polymers were precipitated in methanol (100 mL) and filtrated. The precipitates were purified by a Soxhlet extractor with PE, DCM, and CF as eluent. The polymer was then precipitated in methanol (60 mL) and dried under a vacuum for 24 h before use.

**PBDB-T-2Cl**

PBDB-T-2Cl is synthesized in the same way as SFZn-x. the only difference is BDD-Br and BDT-Sn(Me)_3_ are used in same molar equivalent.

### 2.2. Characterization of Physical Property

The electrochemical linear sweep voltammetry (LSV) was recorded with a computer-controlled electrochemical workstation (Chen Hua CHI600e, Shanghai, China) using a polymer drop-cast film on glassy carbon electrode as the working electrode, platinum wire as the counter electrode and saturated calomel electrode as the reference electrode. 0.1 M Bu_4_NPF_6_ dissolved in acetonitrile was used as the supporting electrolyte. Oxidation processes were measured from 0 V to 1.5 V and reduction processes from 0 V to −1.5 V, scan rate sets to 0.05 V s^−1^. The results were corrected by Fc/Fc^+^.

OSCs were fabricated in the conventional structure of ITO/PEDOT:PSS/polymer:Y6/PDINO/Al. The substrates were cleaned using detergent, deionized water, acetone, and isopropanol every 15 min, and then treated in ultraviolet ozone for 3 min. This is followed by spin coating a thin layer of PEDOT:PSS on the precleaned ITO-coated glass substrates at 4000 rpm for 30 s and then annealed at 150 °C for 15 min. Then, the substrates were transferred into a glovebox. Afterward, for the BHJ devices, the optimized active layer was spin-coated from CHCl_3_ solution with a sum concentration of 15.7 mg mL^−1^ (D:A =1:1.2) at 3200 rpm for 30 s to form an active layer. The fabricated active layer was spin-coated by 1 mg mL^−1^ PDINO solution (methanol as solvent) at 3000 rpm for 30 s. After physical vapor depositing an 80-nm-thick Al electrode on the PDINO interface layer, OSCs were fabricated.

The current density-voltage (J-V) characteristics were measured in the glovebox under AM 1.5 G spectra (100 mW cm^−2^) from a solar simulator (Keithley 2400, Beaverton, OR, USA). The light intensity was calibrated with a 20 mm × 20 mm monocrystalline silicon reference cell. The J-V curves are measured along the forward scan direction from −1.5 to 1.5 V. A solar cell spectral response measurement system (QE-R3011, Kaohsiung, Taiwan) measured the EQE. The light intensity at each wavelength was calibrated with a standard single-crystal Si photovoltaic cell.

## 3. Results and Discussion

**Figure 1:**
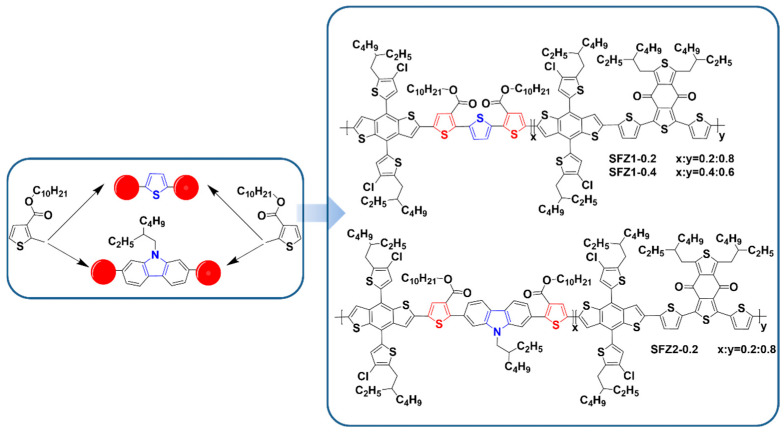
The molecular formula of SFZn-x terpolymers.

Two unfused structures (3T and 2TKZ) are designed and synthesized based on thiophene and 9-(2-ethylhexyl)-9H-carbazole, which linked two decyl thiophene-3-carboxylate (T), respectively [17,18]. Terpolymers SFZn-x (Figure 1) are obtained by randomly and partly replacing BDD units with 3T or 2TKZ in the strong-aggregation BDT-BDD backbones. The n in SFZn-x corresponds to the type of second acceptor units, and x is for the content. For example, SFZ1-0.4 is synthesized by 1 equivalent BDT, 0.6 equivalent BDD, and 0.4 equivalent 3T monomers, while SFZ2-0.2 is obtained by the ratio of 1, 0.8, and 0.2 in BDT, BDD, 2TKZ. The synthesis of the monomers is shown in the Supplementary Information.

**Figure 2:**
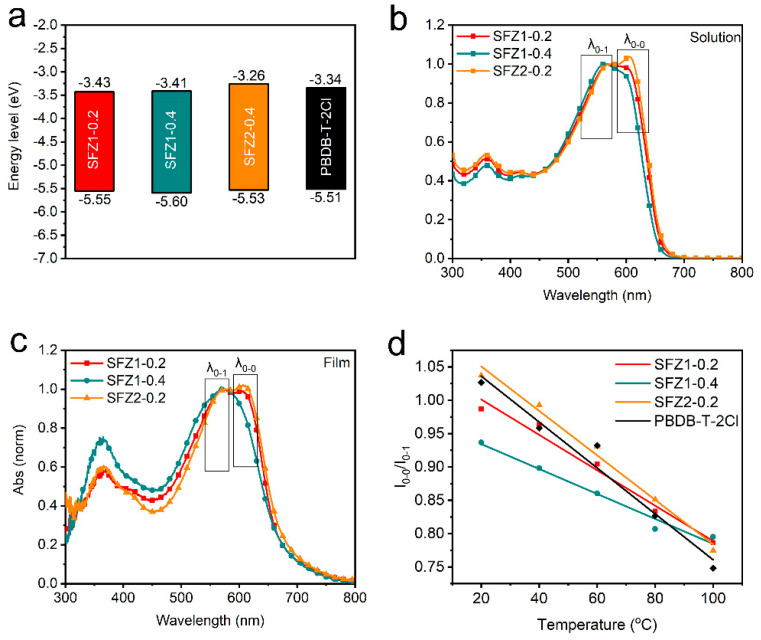
(**a**) Energy levels from LSV curves, absorption in (**b**) solution and (**c**) film, and (**d**) dependence of temperature-I_0-0_/I_0-1_ from UV-vis absorption at different temperatures.

The electrochemical linear sweep voltammetry (LSV) curves of SFZn-x for their onset oxidation and reduction potential (E_onset(OX.)_ and E_onset(RED.)_) are shown in Figure S1. The estimated highest occupied molecular orbital (HOMO) and lowest unoccupied molecular orbital (LUMO) energy levels by E_onset(OX.)_ and E_onset(RED.)_ are presented in Figure 2a and Table 1. Terpolymers and their parent polymer PBDB-T-2Cl reveal similar HOMO energy levels, while LUMO energy levels are slightly upward-shifted for the SFZ2-0.2 than others. This may be related to differences in chemical structure.

The Ultraviolet-visible (UV-vis) absorption spectra of SFZn-x in solution and film are shown in Figure 2b,c. Absorptions possess similar profiles in solution and film, respectively. Film absorptions (λ_onset_, the onset wavelength, at 670~680 nm) are red-shifted compared to solution absorptions (λ_onset_ at 650~665 nm). Similar film absorptions (λ_onset_) imply approaching energy gaps (E_g_) of around 1.84 eV (Table 1). Their strong saddle-shaped absorption at 450~700 nm can be divided into two absorption peaks: the 0-0 (λ_0-0_, wavelength of 0-0 peaks, at ~610 nm) are associated with molecular stacking and the 0-1 (λ_0-1_, wavelength of 0-1 peaks, at ~560 nm) are originated from intramolecular charge transfer. After normalizing according to the intensity of the 0-1 peaks, all absorption spectra expose differences in 0-0 peaks. As the solution (Figure 2b), the 0-0 peak of SFZ1-0.2 is higher than that of SFZ1-0.4, while the 0-0 peak of SFZ2-0.2 is higher than that of SFZ1-0.2. Film absorptions possess consistent trends. The 0-0 peak allows a qualitative comparison of the polymer pre-aggregation, which implies the regulations of molecular aggregation by the type and content of introducing structures in BDT-BDD backbones. In addition, the blue-shifted absorptions of the SFZ1-0.4 in Figure 2b,c imply its weak aggregation [19].

The normalized UV-vis absorption spectra explore aggregation–temperature relationships of terpolymers at 20, 40, 60, 80, and 100 °C (Figure S2). As the temperature increases, the absorption of SFZn-x and PBDB-T-2Cl blue-shifts, while the 0-0 peak decreases. Therefore, the aggregation of terpolymers formed in a low-temperature solution tends to de-aggregate at high temperatures. The blue-shifted 0-1 peak with increasing temperature corresponds to the adjusted planarization of the polymer backbone [20]. Here, the ratio I_0-0_/I_0-1_ of the 0-0 to 0-1 peak intensity and the difference Δλ_0-1_ of λ_0-1_ between 20 and 100 °C is defined to quantify the de-aggregation and adjusted planarization. SFZ1-0.4 indicates the highest polymer backbone adjustability with the highest Δλ_0-1_ in Table 1. Therefore, the 3T structure is favorable for increasing the flexibility of the polymer backbone. As shown in Figure 2d, the I_0-0_/I_0-1_ of the terpolymers decreases quasi-linearly with increasing temperature. At 20 °C, the I_0-0_/I_0-1_ values differed significantly. SFZ2-0.2 indicates the highest I_0-0_/I_0-1_ ratio, exceeding 1, while SFZ1-0.4’s is the lowest. As the temperature increases, the I_0-0_/I_0-1_ ratios of three terpolymers tend to close. At 100 °C, their I_0-0_/I_0-1_ values are almost equal. The aggregation of the SFZn-x terpolymers is regulated by temperature. At low temperatures, the introduced structure dominates the aggregation behavior of the polymers, which leads to different behaviors. In contrast, the polymers obtain enough energy at high temperatures to de-aggregate, and the dominant BDT-BDD structure becomes the main factor affecting the aggregation behavior. Notably, the I_0-0_/I_0-1_ of PBDB-T-2Cl is lower than that of SFZ2-0.2 and higher than that of SFZ1-0.2 and SFZ1-0.4, suggesting enhanced aggregation of 2TKZ and weakened aggregation of 3T in the polymer backbone.

**Figure 3:**
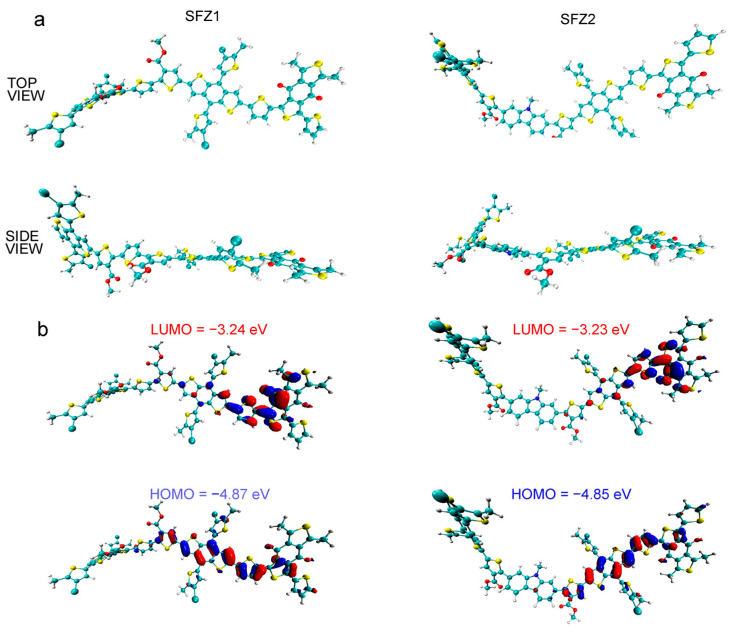
(**a**) The optimized conformations and (**b**) HOMO and LUMO of SFZ1 and SFZ2.

Chain structures play a primary role in determining the physical behaviors of polymers, which often serve as the thermodynamic driving force for structural phase transitions. Thus, the conformations of SFZn-x are simplified as BDT-(3T or 2TKZ)-BDT-BDD with two D-A units, which is to speculate on the modulation of polymer stacking behavior by 3T or 2TKZ under the r^2^SCAN-3c/def2-mTZVPP level using the ORCA 5.0.1 program [21,22,23,24,25,26,27]. Also, a simplified BDT-BDD-BDT-BDD structure, labeled RAW, is explored, which represents the PBDB-T-2Cl chain. And SFZ1 and SFZ2 are reminded as the chains of BDT-3T-BDT-BDD, BDT-2TKZ-BDT-BDD, respectively (Figure S3a). The optimized conformations are shown in Figure 3a. The dihedral angle between the two adjacent conjugated building blocks is one of the most critical issues affecting polymer aggregation [28]. The dihedral angles between the units located in the middle of the conformation are summarized in Table S1 (D). In SFZ1 and SFZ2, their backbones form distorted conformations, and the dihedral angles associated with the ester-substituted thiophene are much higher (∠1 > 20° and ∠2 > 30°) than RAW (∠1, ∠2 ≈ 10°). High ∠1 and ∠2 in SFZ1 and SFZ2 are due to the single-bond internal rotation and hindered rotation crossing ester-substituted thiophene. ∠1 in SFZ1 and SFZ2 are almost equal, while ∠2 in SFZ1 is slightly larger than that in SFZ1, which may be related to the extended space decreasing hindered interaction between two esters. The above results imply that the size of the thiophene or carbazole core unit introduced affects chain conformation. The energy levels and orbital distributions of HOMO and LUMO are shown in Figure 3b. SFZ1 and SFZ2 have approached HOMO and LUMO energy levels, where both SFZ2’s are slightly upshifted compared to that of SFZ1. This result is consistent with the trend estimated by the LSV method. It is worth noting that LUMO and HOMO are distributed primarily in the BDD and BDT units, respectively. The proportion of orbital distributions near the 3T and 2KZ units is small, which suggests that the introduced units do not have much effect on the HOMO and LUMO.

**Figure 4:**
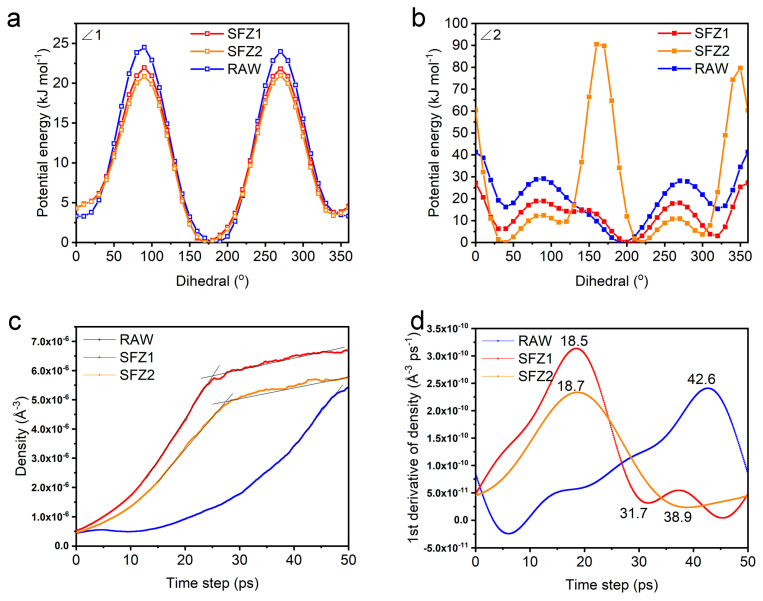
The intramolecular dihedral angles of polymers (∠1 for the dihedral angle between the BDT donor unit and π-bridge, ∠2 for the dihedral angle between the acceptor unit and π-bridge), and the potential energy curve of the internal rotation (**a**) ∠1 and (**b**) ∠2 of polymers. (**c**) Density-time plots of polymer equilibrium processes and corresponding (**d**) derivative curves obtained by molecular dynamics.

A polymer chain possesses semi-flexibility, which characterizes the intra-chain interactions for the most stable conformation persisting along the chain axis. The most common factor that may determine polymer semi-flexibility is internal rotation. The relative potential energy curves of the internal dihedral angles ∠1 and ∠2 are obtained by rigid scanning (Figure S4). The ∠1 curves are saddle-shaped around 0°~360°. The peaks and the troughs are located at ~90°/270° and ~0°/180°, respectively. The ∠2 curves are complex with ~90°/270° peaks and ~30°/200°/310° troughs. The thermodynamic equilibrium based on the relative energy potential (Δε) at troughs defines the static flexibility, and the transition kinetics based on the activation energy (ΔΕ) at peaks defines the dynamic flexibility of polymer chains. The Δε and ΔΕ are summarized in Table S2. For ∠1, SFZ1, SFZ2, and RAW possess similar Δε, but ΔΕ of SFZ1 and SFZ2 are lower; for ∠2, SFZ1 and SFZ2 show slightly higher Δε but significantly lower ΔΕ. Polymer chains will exhibit random coils with high flexibility at low Δε but high rigidity at high Δε. They can easily change their conformation leading to a fluid state. If changing conformations are hindered, polymers are either in the glass or crystalline in the solid state [11]. Therefore, according to Table S2, SFZ2 is the most flexible and least crystallizable, while RAW is the most rigid and crystallizable. However, in Figure 4a,b, the curve of ∠1 is more symmetric at 180° than that of ∠2. ∠2 shows another peak at ~150, which is in descending order of intensity, SFZ2, SFZ1, and RAW. A rigid main chain may limit the conformational behavior of polymers [29]. The high rotational potential energy of SFZ2 results in its conformation fixing in a limited range, exhibiting the highest rigidity and crystallinity, while SFZ1 exhibits the most flexibility and is less crystallizable. Accordingly, the 3T fragment in the polymer increases the flexibility and leads to less crystallizable, while 2TKZ enhances the rigidity and crystallinity. This result is consistent with the fact that SFZ1-0.4 indicates less aggregation but SFZ2-0.2 is more aggregation as demonstrated in UV.

The conformations of polymers were evaluated with molecular dynamics (MD) simulations by the Large-Scale Atomic/Molecular Massively Parallel Simulator (LAMMPS) program [30,31] for the model oligomers 4-D-A-unit (D-A-D-A-D-A-D-A) as RAW, SFZ1, and SFZ2 (Figure S3c). The simulation box contained 30 oligomers distributed randomly in 150 × 150 × 150 Å^3^. The atom file was generated with Auxiliary Tools of Force Field (AuToFF) [32] and the Packing Optimization for Molecular Dynamics Simulations (Packmol) program [33] by All-atom force fields OPLS-AA/L [34,35]. Each model oligomer was simulated at 300 K and 0 atm in the NPT. Figure 4c shows the density curves of the simulation during equilibration at 50 ps. All densities of the model oligomers continued to grow. After rapid growth, the densities of SFZ1 and SFZ2 slowly increased with fluctuations, indicating that the system tends to equilibrate; however, at the end of the simulation, RAW still did not exhibit a clear equilibrium trend. Figure 4c corresponds to the rate of density change. In 50 ps, all oligomers experienced a maximum, with SFZ1 being the first to reach it, followed by SFZ2, and the RAW reached its maximum only at the end. In addition, SFZ1 began to enter relative equilibrium at ~32 ps, SFZ2 was not in relative equilibrium until ~39 ps, and RAW did not exhibit a similar state until the end. The equilibrium of the density profile corresponded to the equilibrium system. The system reached equilibrium from nonequilibrium in the shortest time for SFZ1, indicating that its conformational adjustment was the easiest. This result is in agreement with the previous one. In addition, it also implies that both flexible (3T) and rigid (2TKZ) structures can facilitate the conformational adjustment of polymers.

Based on the above results, the role of the additional unit is mainly to readjust the backbone conformation by internal rotation. For the effect of this internal rotation on the morphology of the blending film, the conformation of the terpolymer with Y6 was explored, which is the most important component of the blending film. The structures of the terpolymers were simplified to BDT-(3T or 2TKZ), and the conformation of donor:accepter (D:A) was explored by the BDT-(3T or 2TKZ):Y6. As a control, BDT-BDD:Y6 was applied as RAW:Y6. The optimized D:A conformations are shown in Figure S5. SFZ1 and SFZ2 exhibited high planarity leading to a face-to-face stacking conformation with Y6. The dihedral angles of the BDT-(3T or 2TKZ) structure in D:A are listed in Table S1 (D:A). Compared to Table S1 (D), both ∠1 and ∠2 of SFZ1 and SFZ2 in the D:A were reduced, showing the tuning of polymer conformation due to intermolecular interactions. Notably, ∠1 and ∠2 of SFZ1 decreased more drastically, suggesting that the SFZ1 is more prone to conformational adjustment by internal rotation. Thus, the 3T fragment is flexible and 2TKZ enhances the rigidity. This is consistent with the rigidity scan results. Among D:A, SFZ1 receives the maximum ∠2, while SFZ1 and RAW can be adjusted to almost planar positions, corroborating that the rigidity of 2TKZ mainly comes from ∠2.

In addition, an atomic force microscope (AFM) was used to explore the SFZn-x:Y6 blends. The surface morphology of the SFZn-x:Y6 blending film is shown in Figure S6. SFZ1-0.2:Y6 still had a high root mean square roughness (S_q_), while SFZ1-0.4:Y6 indicated a flat surface with the lowest S_q_. Therefore, the flexible 3T units in polymers could effectively reduce chain aggregation, which may prevent leading to an over-rugged morphology. SFZ2-0.2:Y6 also showed high S_q_. Due to the more extended chain and low Δε and ΔΕ of carbazole, it was easy to adjust the conformation of carbazole in a limited range, and the more extended area along the chain also facilitates molecular stretching, which would also be beneficial for the optimization of the morphology. Thus, reasonable type and the content of the second unit in polymers can regulate the morphology of the blending film.

**Figure 5:**
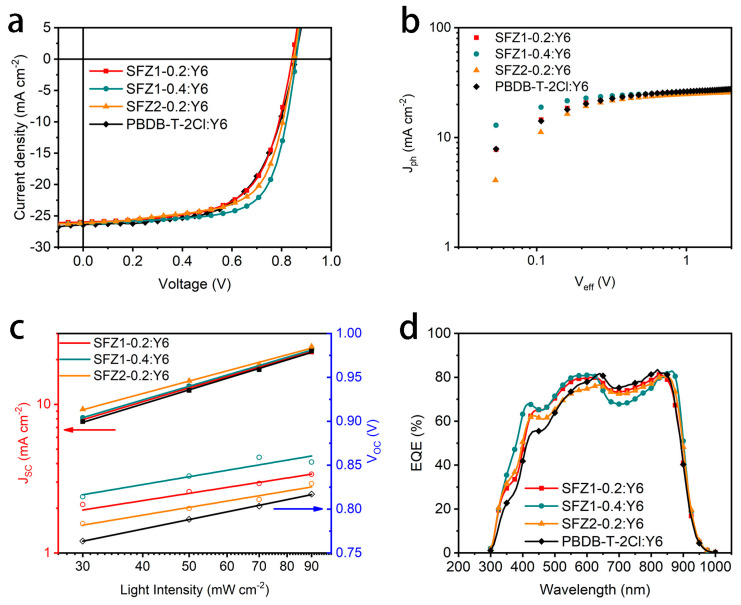
(**a**) J-V curves, (**b**) J_ph_-V_eff_ curves, (**c**) light intensity dependence of J_SC_ and V_OC_, (**d**) EQE spectra of Y6-based OSCs.

The SFZn-x:Y6 and PBDB-T-2Cl:Y6 OSC performances are explored by conventional devices (ITO/PEDOT:PSS/polymer:Y6/PDINO/Al). The current density-voltage (J-V) curves and the corresponding parameters of the devices are shown in Figure 5a and Table 2, respectively. All obtained terpolymer-based devices obtain an open-circuit voltage (V_OC_) > 0.84 V, a short-circuit current density (J_SC_) > 25 mA cm^−2^, and a fill factor (FF) > 63%, resulting in a PCE over 13.85%. Their FF and PCE are higher than that of PBDB-T-2Cl:Y6. Moreover, with the same designed rate (x = 0.2) of the terpolymers, SFZ2-x:Y6 devices obtain higher results (V_OC_, FF) than SFZ1-x:Y6. SFZ1-0.4:Y6 exposes highest performance (V_OC_ = 0.86 V, J_SC_ = 26.13 mA cm^−2^, FF = 69.81%, and PCE = 15.66%). Notably, the FF of OSC improved from 63.37% to 69.81%. It is well known that FF is mainly determined by the device morphology, which is significantly impacted the molecular geometries of donors and acceptors [36]. Thus, the additional flexible and rigid structure can modulate the aggregation and stacking properties of polymers [37], optimizing the morphology of the blending film and effectively enhancing the photovoltaic performance of the corresponding OSCs.

Moreover, the OSC processes, such as exciton dissociation, charge collection, and carrier recombination, were investigated based on SFZn-x:Y6 devices. The J_ph_—V_eff_ relationship and the resulting exciton dissociation efficiency (P_diss_) and charge collection efficiency (P_coll_) are shown in Figure 5b and Table S3. All SFZn-x:Y6-based devices can obtain a P_diss_ close to 0.96. Thus, the additional flexible and rigid structures do not significantly decrease the exciton dissociation process in blends. In addition, SFZn-x:Y6-based devices obtained P_coll_ close to 0.8, which may be related to the fact that the introduced unfused units promote intermolecular interactions and lead to a more efficient intermolecular charge transfer process. The light intensity dependence of SFZn-x:Y6-based devices (J_SC_-I and V_OC_-I) and the resulting α and η are shown in Figure 5c and Table S3. An α close to 1 corresponds to a low bimolecular recombination tendency, while low values of η are associated with low single-molecule or defect recombination. The SFZ1-x:Y6-based device exposes the higher α and lower η, indicating that limited bimolecular, monomolecular, or defect recombination would have occurred during the carrier migration. The above results are similar to the higher J_SC_ of SFZ1-x:Y6-based devices.

The external quantum efficiency (EQE) spectra indicate the photoelectric conversion of the blending films for different wavelength photons (Figure 5d and Table S3). High EQE appears in the wavelength range of 350~950 nm, whose corresponding integral current density (J_EQE_) is about 24~25 mA cm^−2^. The SFZ1-0.4 device exposes the highest EQE in the 350~650 nm corresponding to the highest J_EQE_, consistent with the highest J_SC_ obtained from the J-V curve. Therefore, all devices can achieve efficient photovoltaic conversion. In addition, the electron (μ_e_^−^) and hole (μ_h_^+^) mobilities of the SFZn-x:Y6 devices are measured by the space charge limited current (SCLC) (Figure S7 and Table S3). All μ_e_^−^ of the SFZn-x:Y6 devices reach the order of 10^−4^ cm^2^ V^−1^ s^−1^, while SFZ1-x:Y6 devices indicate μ_h_^+^ at 10^−5^ cm^2^ V^−1^ s^−1^. with higher μ_e_^−^ than μ_h_^+^. Moreover, the SFZ2-0.2:Y6 device has both μ_e_^−^ and μ_h_^+^ at 10^−4^ cm^2^ V^−1^ s^−1^ resulting in a μ_e_^−^/μ_h_^+^ approaching 1, indicating a more balanced electron and hole transport in the device, which implies the impact of additional unit selection and design on performance.

## 4. Conclusions

In conclusion, introducing the flexible and rigid structure into the D-A polymer backbone can efficiently regulate the aggregation and stacking behavior to optimize the BHJ active layer. In polymer chains, the conformation of flexible segments can be easily adjusted by low-energy intramolecular rotation. Although the conformation of rigid chain segments is hard to convert, they may appear in a limited range of low-energy intramolecular rotations to facilitate local conformational adjustment and achieve optimization of the molecular aggregation. Such a strategy can regulate precise optimization by controlling the type and content of the introduced structure. For structures designed from thiophene and carbazole, terpolymer-based OSCs can obtain high V_OC_ (0.84~0.86 V), J_SC_ (25~26 mA cm^−2^), and FF (63~70%), resulting in PCE (13.85~15.66%).

## Figures and Tables

**Table 1:** Optical and electrochemical properties of SFZn-x terpolymers.

Polymer	HOMO (eV) ^a^	LUMO (eV) ^a^	λ_onset_ (nm) ^b^	λ_onset_ (nm) ^c^	E_g_ (eV) ^c^	Δλ_0-1_ (nm) ^d^
SFZ1-0.2	−5.55	−3.43	659	672	1.85	24
SFZ1-0.4	−5.60	−3.41	651	677	1.83	30
SFZ2-0.2	−5.53	−3.26	662	676	1.83	22

^a^ Measured from LSV, where E_HOMO/LUMO_ (eV) = − (E_onset(OX./RED.)_ − E_Fc_^+^_/Fc_ + 4.8), E_Fc_^+^_/Fc_ = 0.36 V. ^b^ Measured from solution state. ^c^ Measured from film state and estimated by E_g_ (eV) = 1240/λ_onset_. ^d^ Δλ_0-1_ (nm) = λ_0-1_ (20 °C) − λ_0-1_ (100 °C).

**Table 2:** Photovoltaic performance of Y6-based OSC under AM 1.5G illumination.

Polymer	V_OC_ (V)	J_SC_ (mA cm^−2^)	FF (%)	PCE_max_ (PCE_ave_) (%) ^a^
SFZ1-0.2	0.84	25.97	63.37	13.85 (13.45)
SFZ1-0.4	0.86	26.13	69.81	15.66 (15.30)
SFZ2-0.2	0.85	26.07	65.53	14.53 (14.09)
PBDB-T-2Cl	0.83	25.96	62.76	13.54 (13.33)

^a^ Average of 10 devices.

## Data Availability

The original contributions presented in this study are included in the article/Supplementary Materials. Further inquiries can be directed to the corresponding author.

## Supplementary Materials

The following supporting information can be downloaded at https://www.mdpi.com/article/10.3390/polym17020248/s1: Figure S1: LSV curves of SFZn-x. Oxidation processes were measured from 0 V to 1.5 V and reduction processes from 0 V to −1.5 V; Figure S2: The UV-vis absorption of (a–c) SFZn-x and (d) PBDB-T-2Cl in chlorobenzene solution at different temperatures; Figure S3: Simplified structures of the polymers, whose structures are simplified to oligomers, and the alkyl group are reduced to methyl groups. (a) 2-D-A-unit D-A-D-A and the intramolecular dihedral angles of polymers (∠1 for the dihedral angle between the BDT donor unit and π-bridge, ∠2 for the dihedral angle between the accepter unit and π-bridge), (b) D-A and the intramolecular dihedral angles, and (c) 4-D-A-unit D-A-D-A-D-A-D-A; Figure S4: The potential energy curves of intramolecular dihedral angles of polymers in Figure S3b (a) RAW, (b) SFZ1, and SFZ2; Figure S5:The conformations of SFZ1:Y6 and SFZ2:Y6; Figure S6: Surface AFM images of SFZn-x:Y6 blending films; Figure S7: J^1/2-V^ curves by SCLC method for Y6-based devices, (a) for electron mobilities and (b) for hole mobilities. Table S1: Dihedral angles in the D and D:A conformations; Table S2: The relative energy potential (Δε) and the activation energy (ΔΕ) based on potential energy curves in Figure S4; Table S3. Photovoltaic performance of the Y6-based devices.
