# Supplementary material for: Effects of Additional Flexible and Rigid Structure on BDT-BDD Terpolymer and the Performance of Organic Solar Cells
Source: Polymers (Basel). 2025 Jan 20;17(2):248. doi: 10.3390/polym17020248 (PMC11769530; doi:10.3390/polym17020248)
Supplement: Supplementary file 1 [file polymers-17-00248-s001.zip › polymers-3406393-supplementary.pdf]

# Supporting Information

## Effects of Additional Flexible and Rigid Structure on BDT-BDD Terpolymer and the Performance of Organic Solar Cells

**Xin Jing [1,2,3], Xuebing Li [3], Yong Zhao [2], Quanliang Wang [2], Xiao Kang [2], Xiaojie Liu [2], Aziz Saparbaev [4], Feng Li [1,*] and Mingliang Sun [2,*]**

[1] Analytical and Testing Center, Qingdao University of Science and Technology, Qingdao 266042, China; jingxin@stu.ouc.edu.cn
[2] School of Materials Science and Engineering, Ocean University of China, Qingdao 266100, China; y.zhao1972@foxmail.com (Y.Z.); wql@stu.ouc.edu.cn (Q.W.); kangxiao1994@126.com (X.K.); liuxiaojie19921005@163.com (X.L.)
[3] Chemistry Department, Paderborn University, 33098 Paderborn, Germany; lixuebing2121@163.com
[4] Institute of Ion-Plasma and Laser Technologies, Uzbekistan Academy of Sciences, 33, Durmon yuli,
Tashkent 100125, Uzbekistan; saparbaevaziz83@gmail.com
* Correspondence: lifeng02@qust.edu.cn (F.L.); mlsun@ouc.edu.cn (M.S.)

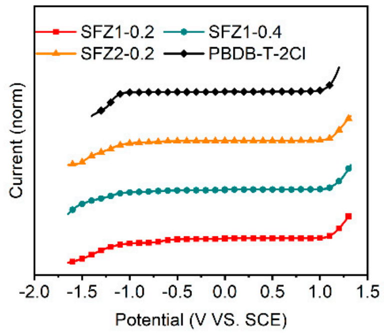


**Figure S1.** LSV curves of SFZn-x. Oxidation processes were measured from 0 V to 1.5 V and reduction processes from 0 V to -1.5 V.

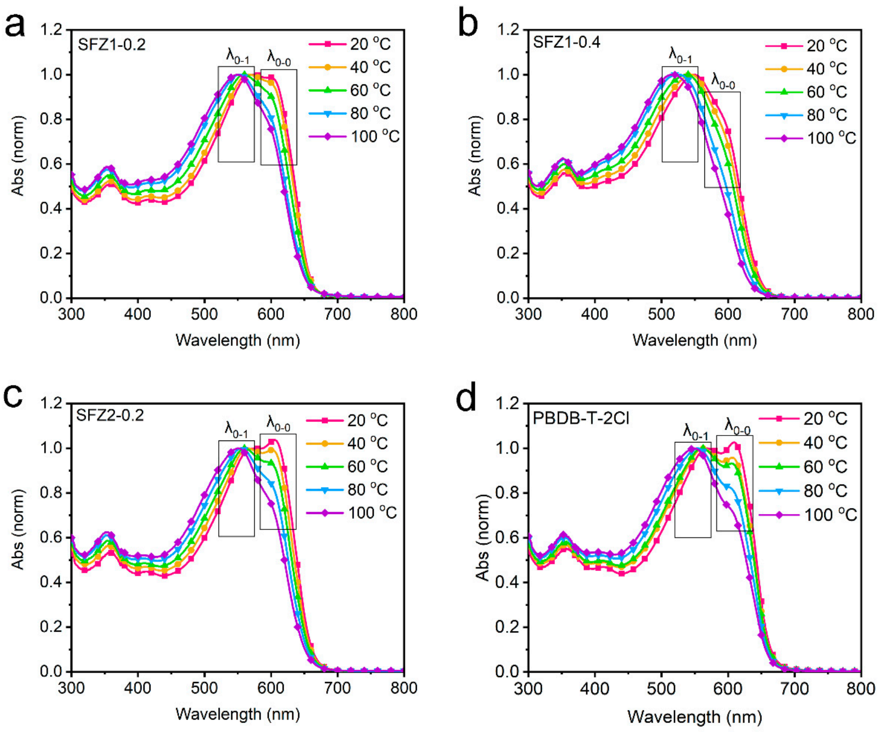


**Figure S2.** The UV-vis absorption of (a-c) SFZn-x and (d) PBDB-T-2Cl in chlorobenzene solution at different temperatures.

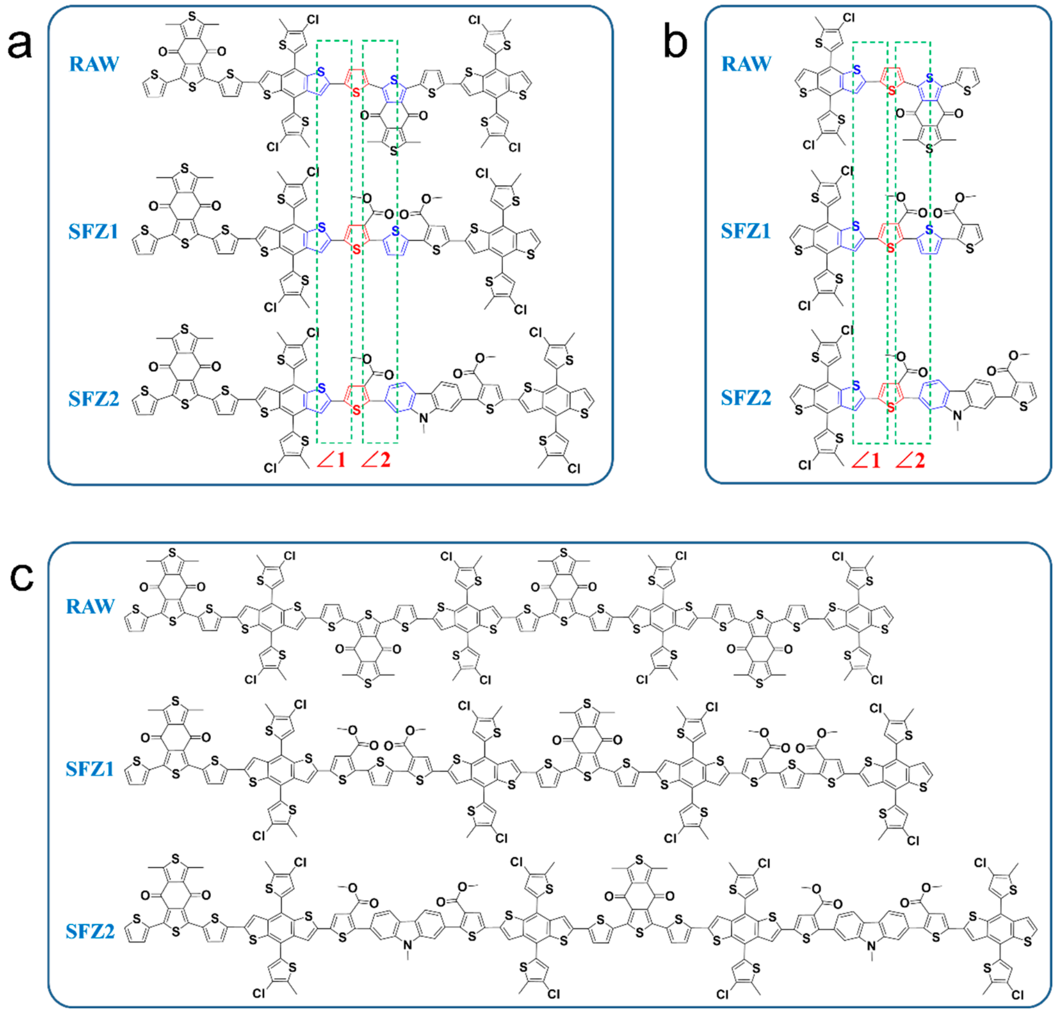


Figure S3. Simplified structures of the polymers, whose structures are simplified to oligomers, and the alkyl group are reduced to methyl groups. (a) 2-D-A-unit D-A-D-A and the intramolecular dihedral angles of polymers (∠1 for the dihedral angle between the BDT donor unit and π-bridge, ∠2 for the dihedral angle between the accepter unit and π-bridge), (b) D-A and the intramolecular dihedral angles, and (c) 4-D-A-unit D-A-D-A-D-A-D-A.

Table S1. Dihedral angles in the D and D:A conformations

| **D** | **∠1 (º)[a]** | **∠2 (º)[a]** | **D:A** | **∠1 (º)[b]** | **∠2 (º)[b]** |
|---|---|---|---|---|---|
| **SFZ1** | 24.78 | 37.38 | **SFZ1:Y6** | 17.43 | 1.16 |
| **SFZ2** | 25.12 | 34.69 | **SFZ2:Y6** | 18.35 | 26.48 |
| **RAW** | 7.35 | 11.18 | **RAW:Y6** | 24.15 | 2.26 |

a As shown in Figure S3a, obtaining from Figure 3a.

b Obtaining from Figure S5.

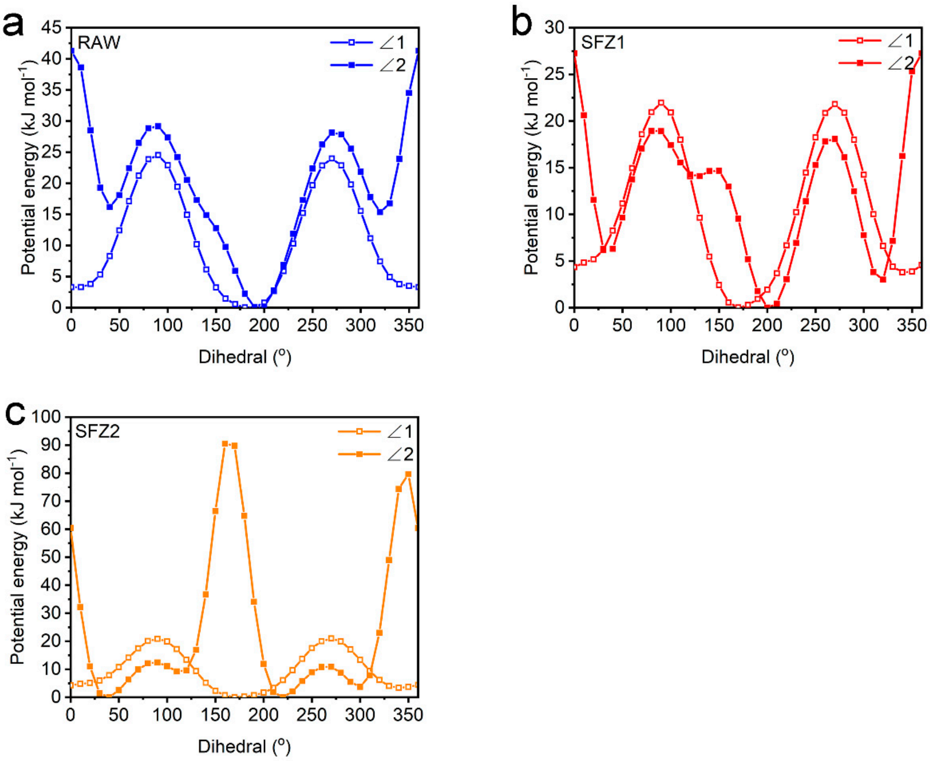


Figure S4. The potential energy curves of intramolecular dihedral angles of polymers in Figure S3b (a) RAW, (b) SFZ1, and SFZ2.

Table S2. The relative energy potential (Δε) and the activation energy (ΔE) based on potential energy curves in Figure S4.

| | **SFZ1** | | **SFZ2** | | **RAW** | |
|---|---|---|---|---|---|---|
| | **∠1** | **∠2** | **∠1** | **∠2** | **∠1** | **∠2** |
| **Δε (kJ mol$^{-1}$)** | 4.34 | 6.30 | 4.27 | 0.00 | 3.29 | 16.18 |
| **ΔE(kJ mol$^{-1}$)** | 21.95 | 18.94 | 20.85 | 12.43 | 24.49 | 29.16 |

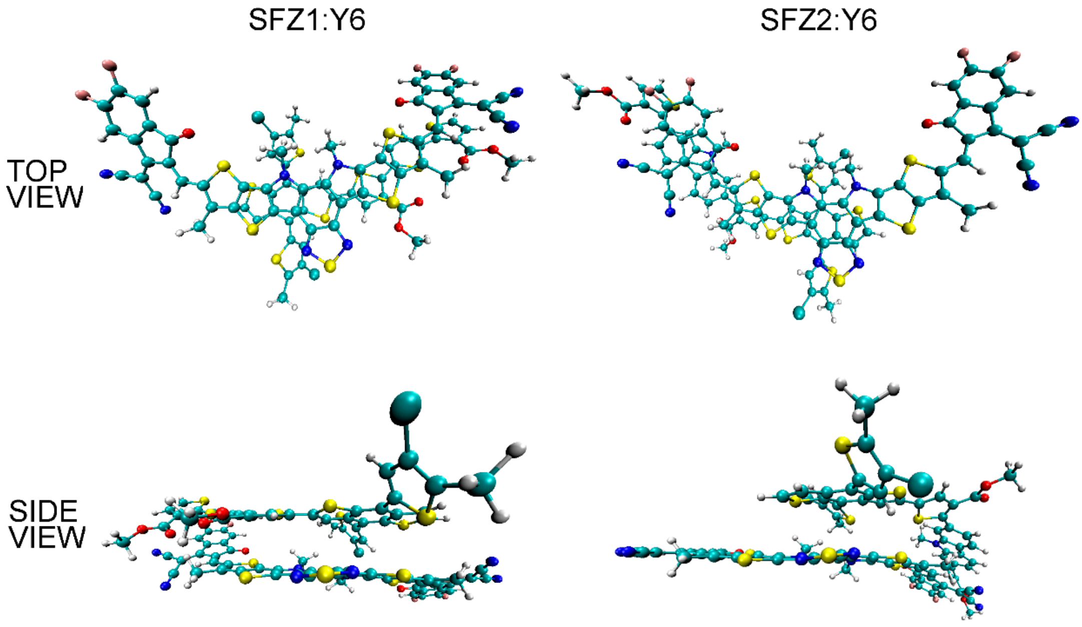


Figure S5 The conformations of SFZ1:Y6 and SFZ2:Y6.

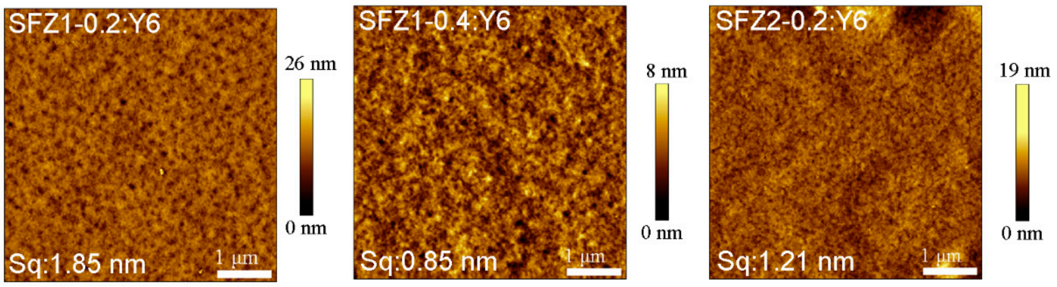


Figure S6 Surface AFM images of SFZn-x:Y6 blending films.

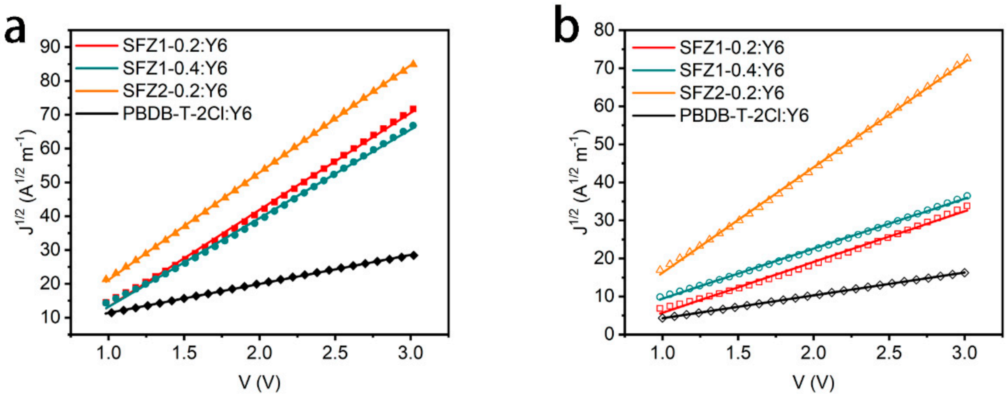


**Figure S7.** $J^{1/2}$-V curves by SCLC method for Y6-based devices, (a) for electron mobilities and (b) for hole mobilities.

Table S3. Photovoltaic performance of the Y6-based devices.

| **Polymer** | $\mathbf{P_{diss}}$ | $\mathbf{P_{coll}}$ | $\boldsymbol{\alpha}$ | $\boldsymbol{\eta}$ | $\mathbf{J_{EQE}}$ (mA cm$^{-2}$) | $\boldsymbol{\mu_e^-}$ (cm$^2$ V$^{-1}$ s$^{-1}$) | $\boldsymbol{\mu_h^+}$ (cm$^2$ V$^{-1}$ s$^{-1}$) | $\boldsymbol{\mu_e^-/\mu_h^+}$ |
|---|---|---|---|---|---|---|---|---|
| **SFZ1-0.2** | 0.961 | 0.780 | 0.96 | 1.19 | 24.81 | $2.75\times10^{-4}$ | $5.97\times10^{-5}$ | 4.615 |
| **SFZ1-0.4** | 0.963 | 0.813 | 0.95 | 1.29 | 25.03 | $2.30\times10^{-4}$ | $5.77\times10^{-5}$ | 3.986 |
| **SFZ2-0.2** | 0.959 | 0.751 | 0.87 | 1.27 | 24.45 | $3.46\times10^{-4}$ | $2.57\times10^{-4}$ | 1.346 |
| **PBDB-T-2Cl** | 0.965 | 0.765 | 0.97 | 1.59 | 24.43 | $2.47\times10^{-5}$ | $1.21\times10^{-5}$ | 2.041 |
